# Methadone Versus Buprenorphine/Naloxone: Treatment Outcome Differences Among Individuals Receiving Medications for Opioid Use Disorders in Rural Treatment Settings—A 1‐Year Retrospective Cohort Study

**DOI:** 10.1111/jrh.70219

**Published:** 2026-09-28

**Authors:** Anna MacCamy, Lisa Khairy, Tara Wasser, Gurkanwal Gill, Leslie Kenefick, Alex Schmidt, Nicole Akana, Luke Hall, Kibeom Kwon, Nicole Limberg, Kirsi Kirk‐Lewis, Megan Lewis, Matt Owen, Sterling M. McPherson, André Q. Miguel

**Affiliations:** ^1^ Elson S. Floyd College of Medicine Washington State University Spokane Washington USA; ^2^ Analytics and PsychoPharmacology Laboratory (APPL) Washington State University Spokane Washington USA; ^3^ Oregon Recovery & Treatment Center (ORTC) Eugene Oregon USA

**Keywords:** buprenorphine, methadone, opioid agonist treatment, retrospective study, rural health

## Abstract

**Background:**

Medication for opioid use disorder (MOUD) is the gold standard of treatment for opioid use disorder (OUD). Methadone and buprenorphine have shown to be the most effective MOUD, yet little is known about the compared efficacy when treating OUD in community‐based rural treatment settings. This study aims to explore treatment outcome differences among patients initiating methadone or buprenorphine/naloxone in community‐based rural treatment programs.

**Methods:**

Data for this 1‐year retrospective cohort study were extracted from electronic medical records of 561 individuals initiating treatment between January 2019 and December 2019 at four MOUD clinics located in rural Oregon. Between‐group comparisons were conducted using chi‐square for dichotomous variables and ANOVA for continuous variables. Kaplan Meier and generalized estimating equations were used to determine treatment retention and opioid use over time.

**Results:**

Of the 561 patients, 74.5% were dispensed methadone while 25.5% were prescribed buprenorphine/naloxone. Those dispensed methadone were more likely to be unemployed (57.7% vs. 44.8%, *p* = 0.01) and diagnosed with severe OUD (93.5% vs. 83.9%, *p* = 0.04). There were no significant group differences on other treatment intake demographics nor opioid use or treatment retention over a 12‐month period. Clinic site was a significant predictor of proportion of negative urine tests.

**Conclusion:**

Both methadone and buprenorphine/naloxone performed comparably well in reducing opioid use and retaining patients in treatment. In comparison to medication type, clinical site and time in treatment had a larger impact on participant outcomes. These findings suggest that the use of both methadone and buprenorphine/naloxone should be encouraged in the context of rural MOUD treatment programs.

## 1 Introduction

In 2024, over 80,000 Americans died from drug‐related overdoses, with approximately 60% involving opioids [1]. While opioid‐related deaths have declined nationally, the geographic distribution of these reductions has not been uniform [1]. Over the past several decades, rates of overdose deaths involving natural, semisynthetic, and synthetic opioids have remained disproportionately higher in rural regions than in urban regions [2]. This disparity is partially attributable to significant healthcare barriers in rural communities [3, 4] including limited number of MOUD clinics [5], a lack of addiction providers [6, 7], transportation challenges [8], and stigma surrounding addiction [9, 10]. Consequently, individuals in rural areas often travel longer distances to access opioid treatment programs, a factor associated with lower adherence to medications for opioid use disorder and higher treatment dropout rates [11, 12].

MOUD—particularly methadone and buprenorphine/naloxone—represent the gold standard for opioid use disorder treatment, reducing withdrawal, opioid use, and overdose [13, 14]. Recent studies have demonstrated comparable effectiveness of methadone and buprenorphine/naloxone in reducing opioid use, with methadone associated with higher treatment retention and increased treatment participation [15, 16, 17].

Despite this evidence, the generalizability of these findings to rural populations remains unclear. Majority of existing studies were conducted in urban or suburban settings or did not report the geographic composition of their samples, limiting their applicability to rural community‐based MOUD programs that face distinct access and resource constraints. This retrospective cohort study compares the effectiveness of methadone versus buprenorphine/naloxone in reducing opioid use and promoting treatment retention among patients in rural Oregon.

We hypothesized that buprenorphine/naloxone would be associated with comparable or greater treatment retention and proportion of negative urine tests relative to methadone among patients in rural community‐based MOUD programs, given that access and transportation barriers unique to rural settings may attenuate methadone's retention advantage observed in previous studies.

## 2 Methods

### 2.1 Design

One‐year retrospective cohort study across four community‐based MOUD clinics located in rural areas of Oregon.

### 2.2 Study Setting

The study was implemented in collaboration with four Oregon Recovery and Treatment Centers, which provide affordable methadone and buprenorphine/naloxone treatments to rural communities (defined here as having populations under 100,000). Each clinic operated independently and was staffed by a team of medical providers, counselors, and administrative staff trained in substance use disorder treatment.

### 2.3 Sample

We reviewed electronic health records from patients who began treatment between January 1 and December 31, 2019. Inclusion criteria included initiation of either methadone or buprenorphine/naloxone treatment in one of these four clinics during this time frame. Treatment options were selected based on shared decision making between the patient and the provider. Given the nature of this study, there were no exclusion criteria.

### 2.4 Data Collection

Sociodemographic information was collected during intake across all clinical sites by treatment staff, along with self‐reported histories of infectious diseases (e.g., HIV and hepatitis). Diagnoses of OUD and psychiatric comorbidities (e.g., PTSD, depression, and anxiety) were based on DSM‐5 criteria. Monthly urinalyses for the 12‐month study period were used to track opioid, methamphetamine, THC, benzodiazepine, and cocaine use. In addition to substance use, the clinics documented treatment adherence, appointment attendance, and disciplinary actions during care. These data were collected as part of routine clinical care and subsequently extracted retrospectively from the electronic medical records by a staff member of Oregon Recovery and Treatment Centers for the purpose of this study.

### 2.5 Outcomes

Primary outcomes were opioid use and treatment retention. Opioid use was assessed using monthly urine toxicology screens and defined as submitting a urine sample with a negative opioid screen in a given month. Treatment retention was measured as the number of days from intake to treatment dropout. Dropout was defined as a 30‐day lapse in treatment attendance without a documented transfer or planned discharge.

### 2.6 Statistical Analysis

Baseline differences were assessed using ANOVA (for continuous variables) and chi‐square tests (for categorical variables). Generalized estimating equations (GEE) were employed to analyze longitudinal changes in opioid use, adjusting for covariates including age, sex, site, OUD severity, and employment. Site, OUD severity, and employment were included in the model given that these variables differed statistically by treatment conditions. Urinalysis results were coded as 1 (positive) or 2 (negative); missed visits were coded as missing. Kaplan–Meier survival analysis was used to assess retention, with treatment condition as the primary predictor. All analyses were conducted using R version 4.0.2, with a significance level of 0.05. Subgroup analyses were also conducted by clinic site and sex to identify potential differential treatment effects.

### 2.7 Missing Data

To comply with the intention‐to‐treat paradigm, we addressed missing urinalysis data using three approaches: (1) default (listwise deletion), (2) multiple imputation using Rubin's rules [18], and (3) worst‐case scenario (assuming all missing samples were opioid‐positive). These sensitivity analyses allowed us to test the robustness of our findings across varying assumptions about patient behavior and data mechanisms.

### 2.8 Ethics

This study was reviewed by the Institutional Review Board and determined to be exempt from further review. Prior to analysis, the dataset was reviewed and limited to study variables that did not contain identifiable information.

## 3 Results

### 3.1 Intake Characteristics

As can be seen in Table 1, of the 561 patients 74.5% received methadone. The average age was 36.7 years (SD = 10.7). The majority were White (89.5%) and male (54.5%). Many reported legal issues (26.2%) and unemployment (54.4%), while 5.3% were unhoused at intake. Nearly all (91.1%) were diagnosed with severe OUD. Urinalysis at intake revealed high rates of opioid (66.3%), methamphetamine (49.4%), and THC (40.6%) use. Benzodiazepine and cocaine positivity rates were lower at 8.4% and 2.7%, respectively. Comorbid psychiatric diagnoses included depression (24.8%), anxiety (33.7%), and PTSD (16.2%). Additionally, 16.2% reported hepatitis and one person reported HIV.

**TABLE 1:** Intake sociodemographic and clinical profile by treatment condition.

	Total sample (*n* = 561)	Methadone (*n* = 418, 74.5%)	Buprenorphine/naloxone (*n* = 143, 25.5%)	*p*‐value
Treatment site, *n* (%)				
Clinic 1	103 (18.4)	97 (23.2)	6 (4.2)	**0.001** *
Clinic 2	190 (33.9)	150 (35.9)	40 (28.0)	
Clinic 3	163 (29.1)	113 (27.0)	50 (35.0)	
Clinic 4	105 (18.7)	58 (13.9)	47 (32.9)	
Age, mean (SD)	36.7(10.7)	37.1 (11.1)	35.6 (9.5)	0.158
Biological sex, *n* (%)				
Male	306 (54.5)	239 (57.2)	67 (46.9)	0.109
Sexual orientation, *n* (%)				
Heterosexual	519 (92.5)	390 (93.3)	129 (90.2)	0.575
Homosexual	10 (1.8)	9 (2.2)	1 (0.7)	
Bisexual	15 (2.7)	11 (2.6)	4 (2.8)	
Other	4 (0.7)	2 (0.5)	2 (1.4)	
Race, *n* (%)				
White	502 (89.5)	377 (90.2)	125 (87.4)	0.799
Native American	21 (3.7)	14 (3.3)	7 (4.9)	
Hispanic	24 (4.3)	20 (4.8)	4 (2.8)	
African‐American	5 (0.9)	4 (1.0)	1 (0.7)	
Asian	1 (0.2)	1 (0.2)	0 (0.0)	
Alaska Native	1 (0.2)	1 (0.2)	0 (0.0)	
Employment, *n* (%)				
Employed	181 (32.3)	119 (28.5)	62 (43.4)	0.010*
Unemployed	305 (54.4)	241 (57.7)	64 (44.8)	
Disabled	52 (9.3)	43 (10.3)	9 (6.3)	
Student	6 (1.1)	5 (1.2)	1 (0.7)	
Homemaker	12 (2.1)	10 (2.4)	2 (1.4)	
Homeless, *n* (%)	30 (5.3)	20 (4.8)	10 (7.0)	0.372
Legal issues, *n* (%)	147 (26.2)	104 (24.9)	43 (30.1)	0.195
OUD diagnosis, *n* (%)				
Severe	511 (91.1)	391 (93.5)	120 (83.9)	0.040*
Moderate	44 (7.8)	27 (6.5)	17 (11.9)	
Mild	0	0	0	
Positive opioid UA, *n* (%)	372 (66.3)	262 (62.7)	110 (76.9)	0.004*
Positive methamphetamine UA, *n* (%)	277 (49.4)	211 (50.5)	66 (46.2)	0.381
Positive cocaine UA, *n* (%)	15 (2.7)	9 (2.2)	6 (4.2)	0.326
Positive THC UA, *n* (%)	228 (40.6)	164 (39.2)	64 (44.8)	0.327
Positive benzodiazepine UA, *n* (%)	47 (8.4)	35 (8.4)	12 (8.4)	1.000
Depression, *n* (%)	139 (24.8)	100 (23.9)	39 (27.3)	0.371
Anxiety, *n* (%)	189 (33.7)	137 (32.8)	52 (36.4)	0.323
PTSD, *n* (%)	91 (16.2)	64 (15.3)	27 (18.9)	0.108
Hepatitis, *n* (%)	91 (16.2)	70 (16.7)	21 (14.7)	0.849
HIV, *n* (%)	1 (0.2)	0 (0.0)	1 (0.7)	0.554

*Note*: bold and * = p‐value <0.05.

Abbreviations: OUD = opioid use disorder; *n* = sample size; SD = standard deviation; UA = urinalysis.

Methadone recipients were significantly more likely to be unemployed (57.7% vs. 44.8%, *p* = 0.01) and to be diagnosed with severe OUD (93.5% vs. 83.9%, *p* = 0.04). Treatment distribution varied by clinic (*p* = 0.001), with Clinic 1 having the highest methadone use (94.2%) and Clinic 4 the lowest (55.2%).

### 3.2 Opioid Use Outcomes

As can be seen in Table 2, the GEE models revealed no statistically significant difference in opioid use over time between the methadone and buprenorphine/naloxone groups, regardless of how missing data were handled. Time was associated with increasing odds of negative urine screens in the default (OR = 1.11, *p* < 0.001) and imputed models (OR = 1.04, *p* = 0.009) but showed a decline in the worst‐case model (OR = 0.96, *p* < 0.001).

**TABLE 2:** Proportion negative urine tests for opioids over time by treatment condition.

	Default			Multiple imputation			Worst‐case scenario		
	OR	95% CI	*p*‐value	OR	95% CI	*p*‐value	OR	95% CI	*p*‐value
Treatment—buprenorphine/naloxone	0.95	0.68–1.33	0.771	0.90	0.64–1.26	0.542	0.82	0.58–1.15	0.241
Time (month)	**1.11**	**1.08–1.13**	**<0.001**	**1.04**	**1.01–1.06**	**0.009**	**0.96**	**0.94–0.98**	**<0.001**
Treatment by time interaction	0.99	0.945–1.034	0.610	0.99	0.95–1.04	0.811	0.99	0.95–1.04	0.841
Clinic 1	**2.59**	**1.69–3.98**	**<0.001**	**1.98**	**1.21–3.24**	**0.01**	1.21	0.79–1.85	0.384
Clinic 2	**1.61**	**1.13–2.29**	**0.009**	1.34	0.93–1.92	0.115	0.93	0.65–1.32	0.675
Clinic 3	0.99	0.68–1.44	0.950	1.03	0.69–1.53	0.904	0.78	0.53–1.14	0.196
Sex—male	1.13	0.87–1.45	0.374	1.25	0.93–1.69	0.141	1.19	0.92–1.54	0.196
Age	1.01	0.99–1.02	0.336	1.01	0.99–1.02	0.197	1.01	0.99–1.02	0.316
OUD diagnosis—moderate	0.85	0.55–1.32	0.4741	0.75	0.42–1.34	0.343	0.85	0.54–1.34	0.491
Employment—unemployed	1.02	0.76–1.36	0.89	1.00	0.72–1.39	0.998	1.08	0.82–1.44	0.578

*Note*: Reference levels for treatment, clinic, sex, OUD diagnosis, and employment are methadone, clinic 4, female, severe, and employed, respectively. Bold = p‐value <0.05.

Clinic site was a significant predictor of reduced opioid use. Compared to Clinic 4, participants at Clinic 1 (OR = 2.59, *p* < 0.001) and Clinic 2 (OR = 1.61, *p* = 0.009) had higher odds of opioid‐negative results in the default model. In the imputed model, only Clinic 1 showed significantly better outcomes (OR = 1.98, *p* = 0.01).

### 3.3 Treatment Retention

Kaplan–Meier analysis showed no significant difference in retention between methadone and buprenorphine/naloxone (*p* = 0.9). Median days retained in treatment were similar across groups. Most participants remained in treatment for several months, with a small proportion reaching the full 12‐month duration (Figure 1).

**FIGURE 1:**
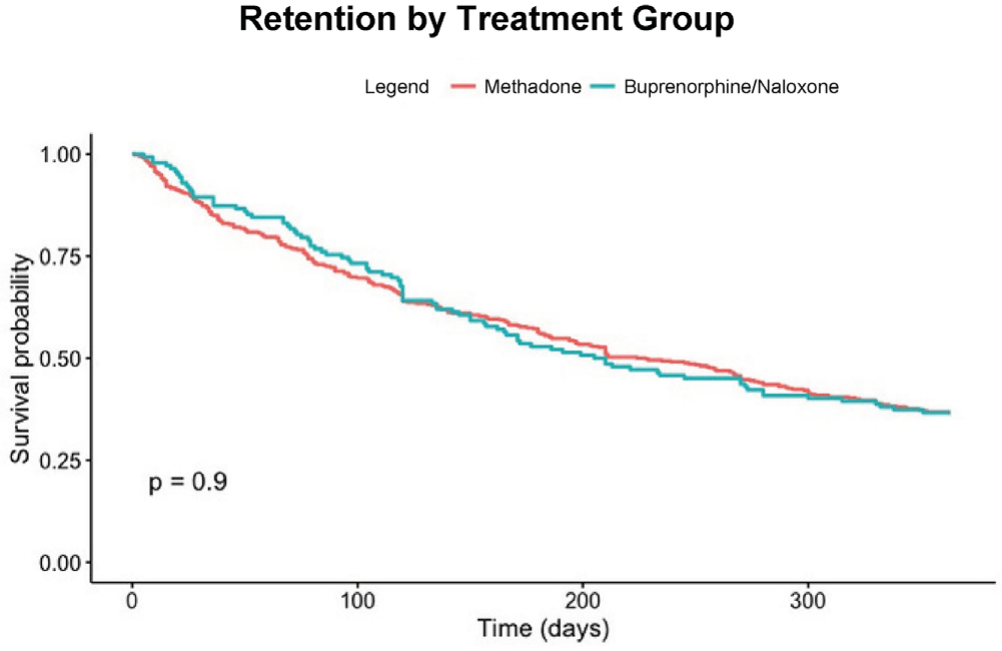
Survival analysis forthe treatment retention outcome.

## 4 Discussion

This study is the first to directly compare outcomes associated with methadone and buprenorphine/naloxone in rural, community‐based MOUD treatment programs. Among more than 500 patients across four clinics in rural Oregon, we found no significant differences between the two medications in reducing opioid use or improving treatment retention. These findings contrast with previous studies reporting higher treatment retention among patients receiving methadone. Overall, our results suggest that methadone and buprenorphine/naloxone are comparably effective treatment options for OUD in rural settings.

Nearly three‐quarters of patients in our sample received methadone, despite both medications being covered by public insurance. This predominance may reflect differences in clinic‐level prescribing practices, patient preferences, or access to medication‐specific services. The substantial variability in methadone prescribing across clinic sites further suggests that local practice patterns and organizational factors may have influenced medication selection. At the time of the study, methadone treatment also generally required daily in‐person dosing and was subject to more restrictive federal regulations than buprenorphine/naloxone, which may have created additional logistical barriers for rural patients. Conversely, established methadone infrastructure or greater provider experience with methadone at some sites may have contributed to its more frequent use. Because our dataset did not capture clinic‐level prescribing protocols, provider preferences, patient treatment preferences, or access‐related factors, we cannot determine the relative contribution of these factors. Future research should examine how clinic‐level practices and rural treatment infrastructure shape medication selection in MOUD programs.

Consistent with prior work [16], longer duration in treatment was associated with reduced opioid use, underscoring the importance of sustained engagement with MOUD. Additionally, treatment site emerged as a significant predictor of reduced opioid use suggesting that clinic‐level or geographic factors may meaningfully influence outcomes in rural settings. However, the absence of data on clinic structure and geography limited further exploration of these differences.

Contrary to our initial hypothesis and prior urban‐focused studies [16, 17], methadone was not associated with higher treatment retention than buprenorphine/naloxone. As mentioned, greater travel burden in rural settings [11, 12] may attenuate the retention advantage observed with methadone elsewhere. Whatever the case, these findings challenge assumptions derived from urban‐centric studies and highlight the need for rural‐specific evidence to inform MOUD delivery in this setting.

### 4.1 Strengths and Limitations

Strengths of this study include its real‐world setting across multiple rural clinics, use of analytic methods incorporating three missing data assumptions, and a large sample size (*n* = 561) for a rural population. The 12‐month follow‐up period supports assessment of sustained outcomes, and inclusion of both methadone and buprenorphine/naloxone‐treated individuals enabled meaningful comparisons.

This study has several limitations. Key clinical information was unavailable, including medication dose, psychosocial support, and driving distance to treatment, limiting our ability to fully control for confounding factors such as patient motivation and comorbid conditions. Opioid use was determined by a single monthly urinalysis, which may not accurately capture opioid use between tests. This measure also does not reflect the fact that total abstinence is not the goal for all patients seeking treatment. The sample was racially homogeneous (89.5% White) and drawn from clinics in a single state (Oregon), limiting generalizability to more diverse rural settings. Additionally, the study period ended in December 2020 and therefore does not capture post‐COVID changes in MOUD delivery, including expanded telemedicine access and updated federal regulations permitting increased take‐home methadone supplies, nor does it reflect subsequent shifts in the drug supply, particularly the emergence of fentanyl and other synthetic opioids.

## 5 Conclusion

Methadone and buprenorphine/naloxone appear comparably effective for reducing opioid use and retaining patients in treatment in rural community‐based MOUD treatment programs. Site‐specific differences and overall time in treatment played a more decisive role in outcomes than medication type. These findings suggest that expanding access to both medications while simultaneously addressing clinic‐specific barriers may be the most effective strategy for improving outcomes in rural OUD treatment.

Further research addressing treatment choice, clinic‐level variability, geographic factors (i.e., access to transportation and commute time) is needed to optimize care delivery in underserved rural areas. Finally, integrating qualitative research to capture patient and provider experiences and expanding data collection to include post‐pandemic treatment trends are essential in informing future policy and program development.

## Funding

No funding was received for this research.

## Conflicts of Interest

The authors declare no conflicts of interest.
